# ATR–FTIR‐Based Direct and Rapid Quantification of Levofloxacin, Degradation and Adulterations via Multivariate Analysis

**DOI:** 10.1002/ansa.70048

**Published:** 2025-10-03

**Authors:** Muhammad Umair Kamal, Raja Adil Sarfraz, Rizwan Ashraf, Muhammad Kashif, Muhammad Imran

**Affiliations:** ^1^ Department of Chemistry University of Agriculture Faisalabad Pakistan; ^2^ Physical to Life Sciences Research Hub, FOCAS Building Technological University Dublin, City Campus Dublin Ireland; ^3^ National Institute for Biotechnology & Genetic Engineering (NIBGE) Faisalabad Pakistan

**Keywords:** adulteration | ATR–Fourier transformed infrared spectroscopy (FTIR) | direct and rapid quantification | levofloxacin (LFX) | principal component analysis (PCA)

## Abstract

The rapid and direct drug quantification is indispensable for quality control units in the pharmaceutical sector to assure safe formulation and delivery of the right medicine to patients. Spectral features of Levofloxacin (LFX) were examined to provide a Fourier transform infrared spectroscopy (FTIR)‐based rapid and pragmatic analytical method for direct quantification of LFX in solid formulations. A chemometric model was developed for frequency region of 1252.39–1218.84 cm^−1^ which is linear over the range of 30%–90% (w/w) with a coefficient of determination (*R* ^2^) 0.995 and meets the requirements of International Council for Harmonization (ICH) and AOAC for the development of the method. The limit of detection (LOD) and limit of quantification (LOQ) were found to 7.616% w/w and 23.079% w/w, respectively. Principal component analysis (PCA) was performed to identify adulteration or degradation of active pharmaceutical ingredients (APIs). It was observed that PC1 and PC2 explained 99.93% and 99.91% of the IR spectral variance respectively. Consequently, this method can be applied for routine analysis of LFX and quality control in pharmaceutical regulatory laboratories.

## 1 Introduction

Fourier transformed infrared spectroscopy (FTIR) is an important instrument in pharmaceutical industries and regulatory bodies for identification of active ingredients in pharmaceutical formulation (API). The frequency of IR spectrum absorbed and their respective absorption intensities refer to particular arrangement of functional groups or chemical structure of pharmaceutical products, and this hypothesis can be employed for the qualitative and quantitative determination of API. Currently, analytical scientists are developing FTIR‐based analytical methods of analysis which are effective, both as qualitative and quantitative measurements for quality control of pharmaceutical products as rapid and cost‐effective tool [1, 2].

ATR–FTIR spectroscopy has an advantage over classical IR transmission spectroscopy for easy sample handling that just needs to place small amount of powder on refractive‐index crystal cell without prior treatments. Moreover, IR spectroscopy is very sensitive to the presence of contaminations or adulterations, showing significant changes by appearance of new bands and/or slight changes in typical bands of spectrum of the test compounds [3, 4]. These slight variations in spectrum could ostensibly be mere in strength, but data processing through chemometric model or multivariate statistical analysis could analyse reliably for their identification. For multivariate analysis, most common statistical tools are principal component analysis (PCA) and partial least squares regression analysis that significantly demonstrate identification of mere changes and their quantification through Cartesian plane prior to PLS regressions [5].

Levofloxacin (LFX) (C_18_H_20_FN_3_O_4_, MW 370.38), an isomer of ofloxacin, is a synthetic broad‐spectrum antibiotic of fluoroquinolone family. LFX once administered targets the bacterial gyrase and topoisomerase IV by binding to their DNA [6]. The compromised dose of drug, whether it is due to lower API or drug degradation due to poor storage, may cause drug resistance and severe side effects [7]. Therefore, the drug regulation for quality and safety is inevitable in healthcare regime for effective disease control and unwanted side effects. All this scenario calls for a rapid, economical and direct analytical method to investigate and regulate quality of medicine [8]. There are multiple spectroscopic and chromatographic methods available for analysis of LFX tablets, but many of these either need extensive sample preparation/extraction, poor selectivity and/or involve the use of hazardous solvents [9, 10]. Besides, majority of chromatographic methods are lengthy, involved costly apparatus and need expertise of the laboratory scientists [11].

The stakeholders nowdays are looking for on‐site, rapid and direct analysis of drugs for efficient analytical services. In this regard, the present study has been undertaken to devise a rapid and direct assay method for the quantification of LFX in solid formulations. Moreover, possible degradation and adulteration were identified through multivariate statistical analysis and quantified through partial least squares regression analysis.

## 2 Materials and Methods

### 2.1 Chemicals and Sample Collection

LFX certified reference material (CRM) standard was purchased from Sigma Aldrich Germany. Commercial samples of different brands having LFX API (Leflox 500 mg, Floxolev 500 mg and Curafloxin 500 mg) were purchased from the local pharmacy in Lahore. A mixture of commonly used different excipients of USP grade (starch powder, avicel, lactose monohydrate and talcum powder) was acquired from a local pharmaceutical in Lahore—Pakistan.

### 2.2 Sample and Calibration Standards Preparation

A range of calibration standards (30%–90%) were prepared by using CRM LFX and diluent (which is mixture in specified ratio of commonly used excipients for LFX formulation) to get the final weight of 300 mg. These prepared solid mixtures of API and excipients of different concentrations are described in Table 1. These (solid/solid) physical mixtures were prepared by weighing of excipient and API in varying proportion to obtain the desire concentration, and homogeneity of the mixtures was carefully handled by thoroughly mixing for maximum time to obtain well‐defined and quantifiable spectra. The test samples (20 tablets of each sample) were separately crushed in mortar and pestle for 10 min to obtain fine powder of homogenous mixture. In order to obtain a regression model, all these calibration standards and test samples were placed directly on the lens of ATR–FTIR.

**TABLE 1:** Preparation of calibration standards for calibration curve.

Sr. no.	Sample name	Excipients (mg)	API (mg)	Gross weight (mg)	% w/w
1	SPL‐1	210	90	300	30
2	SPL‐2	180	120	300	40
3	SPL‐3	150	150	300	50
4	SPL‐4	120	180	300	60
5	SPL‐5	90	210	300	70
6	SPL‐6	60	240	300	80
7	SPL‐7	30	270	300	90

Abbreviations: API, active pharmaceutical ingredient.

Adulteration of test samples was prepared through test samples of LFX and purposefully adulterated by Ofloxacin (OF 1&2) and Ciprofloxacin (CF 1&2). The mixture was thoroughly homogenized, and FTIR spectra were acquired. The forced degradation is a recommended procedure of International Council for Harmonization (ICH) guidelines to conduct stability studies [12]. Test samples were prepared by forced degradation by exposing with different stressed conditions like UV–visible light (LE), heat (TE) and acid stress (AE). LFX tablets (LE 1&2) were exposed to sunlight. The tablets were placed in clean and humid free transparent container for about a day, followed by crushing for FTIR spectra. The LFX tablets (TE 1&2) were placed in oven at 120°C for 6 h. Test samples AE 1&2 were exposed to fumes of acid (0.5 N HCl) for 6 h and samples were dried, crushed and homogenized, and spectra were collected by directly placing on lens of FTIR.

### 2.3 Spectral Acquisition and Data Analysis

All dilutions, including calibration standards (30%–90% w/w) and test samples, were scanned by using Agilent 630‐ATR‐FTIR spectrophotometre. The spectrophotometre is equipped with a temperature‐controlled diamond‐attenuated total reflectance. The FTIR is handled with its micro lab software to acquire the spectra of calibration standards and samples. A small quantity of mixture was directly placed on the ATR‐lens of the instrument at ambient laboratory temperature. The spectra of all prepared calibration standards were acquired in transmission mode (%T) in the spectral range of 4000–400 cm^−1^ at resolution of 2 cm^−1^. Figure S7 shows the transmission spectra which were later converted into absorbance spectra during post processing. The spectral data obtained from ATR–FTIR were post processed by using Microlab Quant Software 5.1, Matblab‐7.8 and essential FTIR software.

### 2.4 Method Development and Validation

The validity of the quantification method was established by complying the guidelines of ICH Q2 (R1), titled ‘The Validation of Analytical Procedures’. The developed method validity is established by performing the described parameters in ICH guidelines; specificity, linearity, accuracy, precision, limit of detection (LOD) and limit of quantification (LOQ) [13].

### 2.5 Specificity

The specificity of developed method was assured through overlay of individual FTIR spectra of pure API with FTIR spectra of excipients. Specificity demonstrates the interaction of impurities, degradants and excipients that could interfere the selectivity of the method. Spectra of each individual excipient were analysed, and the specificity to API was established [14].

### 2.6 Linearity LOD and LOQ

Linearity of the developed method was determined over the concentration range of 30%–90% w/w and was established by measuring the *R* ^2^ value. Linearity of the curve was obtained by visual method in terms of correlation coefficient (*R* ^2^). The slope of this calibration curve (peak area vs. concentration) was used to calculate the LOD and LOQ [15].

The LOD and LOQ were calculated by using the formulas described in ICH guidelines [16]. For LOD = SD × 3.3/*S* and for LOQ = SD × 10/*S*, SD is the standard deviation of the intercept and *S* is the slope of the calibration curve.

### 2.7 Precision

The precision of the ATR–FTIR‐based developed method was gauged up by repeatability and reproducibility (intermediate precision) [17]. According to the ICH guidelines for repeatability and intermediate precision, six determinates of three different concentrations (30%, 50% and 70%) were performed. The results obtained were processed for intraday (repeatability) and inter‐day (reproducibility) relative standard deviation (% RSD).

### 2.8 Accuracy

The accuracy of method was validated through recovery analysis of three levels of LFX: low concentration 80%, medium concentration 100% and high concentration 120%. The concentration of test tablets according to the manufacturer label claim was 76.2%. In order to prepare these 80%, 100% and 120% concentrations of the label claim, the standard and excipients were added to the crushed powder of tablets. The nine determinants were performed by using these concentrations. The results of accuracy measurements in FTIR analysis are presented as ratio of experimental findings versus theoretical values of concentrations [18].

### 2.9 Validation of Method by HPLC‐PDA

Assays of the test samples as well as calibration standard were determined on HPLC by using monograph of US Pharmacopeia, for LFX tablets to validate the results tested by the FTIR. The analysis was carried out on HPLC (Alliance e2689 Waters) equipped with photodiode array detector (2489 Waters). The chromatographic conditions for HPLC analysis were as follows: mobile phase 70% buffer (874 mg of cupric sulphate, 918 mg of l‐isoleucine and 5.94 g of ammonium acetate in 1 L of water) and 30% of methanol. The column used for the analysis was octadecylsilane (4.6 mm × 25 cm; 5 µm).

## 3 Results and Discussions

### 3.1 Spectral Characterization, Wavenumber Selection and Matrix Interference

LFX belongs to quinolone family, having specific chemical structure containing several functional groups, including but not limited to carbonyl group (–CO), ether linkage (–O–) acidic group (–COOH), halogen (–F), aromatic rings, tertiary amines and so forth [19]. The ability of the IR spectroscopy arises due to the fact that the covalent bonds comprising different functional groups have different absorption frequencies. The magnitude of the absorption depends on the moles of functional groups present in the compound which was used to quantify LFX. The IR frequency transmission patterns especially within 1800–800 cm^−1^ reflect functional groups and fingerprint regions through prominent frequency mode as details are given in Table 2. The typical functional group of tertiary amines (C–N) showed frequency absorption at 1350–1320 cm^−1^, whereas ether linkage (–O–) and halogen group (C–F) showed absorption frequency at regions 1080–1020 and 1400–1100 cm^−1^, respectively.

**TABLE 2:** The concentration‐dependent mid‐IR absorption regions of levofloxacin obtained using ATR–Fourier transformed infrared spectroscopy (FTIR).

Sr. no.	Peak value (cm^−1^)	Functional group	Mode of vibrations	Strength
1	801	C–H	Bending	Weak
2	873	Aromatic ring	C–H wagging	Strong
3	1087	C–O	Stretching	Strong
4	1258	C–F	Stretching	Strong
5	1341	C–N	Stretching	Strong
6	1395	O–H	Bending	Strong
7	1517	N–H	Bending	Weak
8	1619	C=C	Stretching	Strong
9	1720	C=O	Stretching	Strong

**FIGURE 1:**
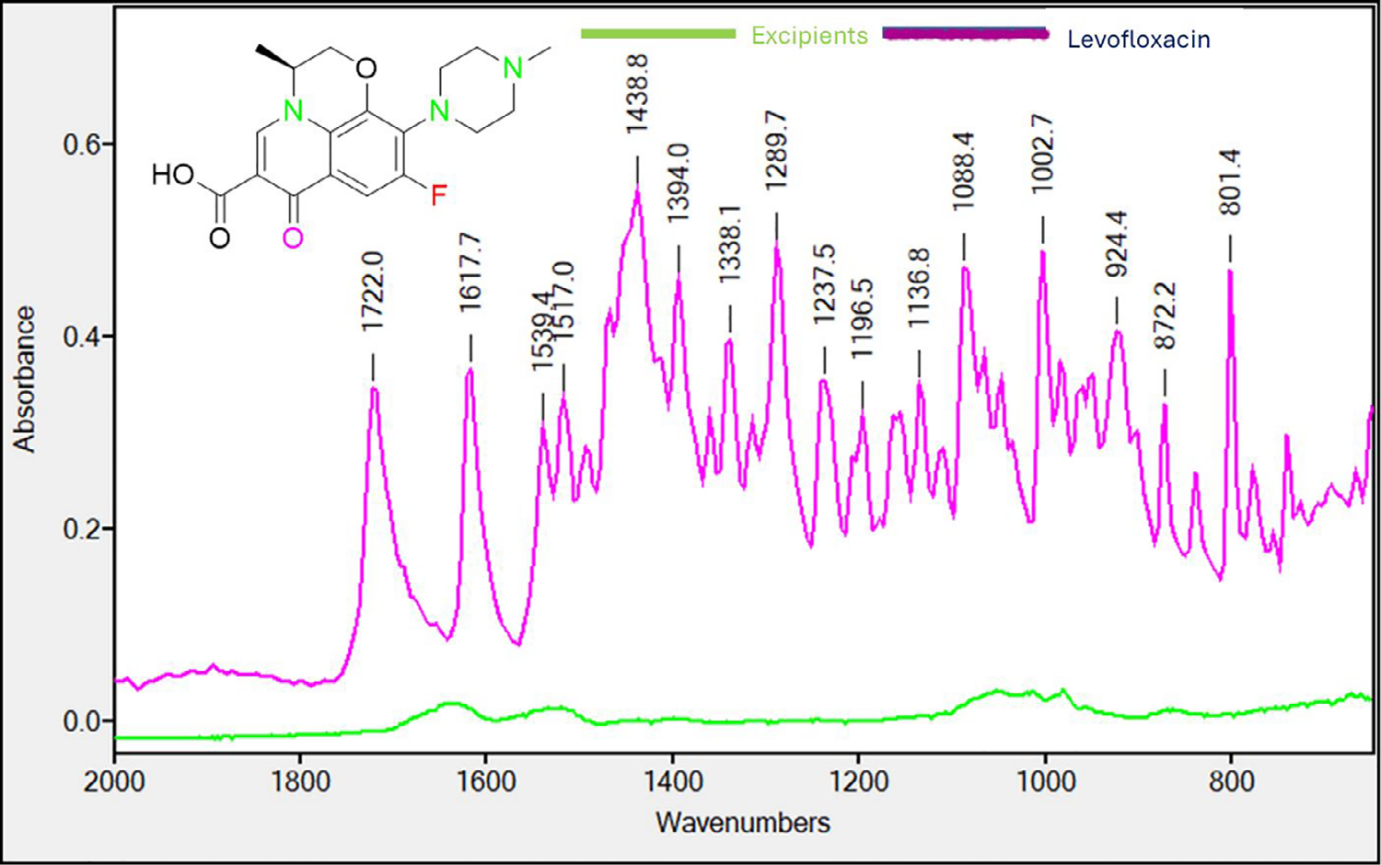
FTIR spectrum of levofloxacin API and excipients in the region of 2000–700 cm^−1^ exhibits no matrix interference.

The spectra when probed for possible interference of excipients with active substance, at the stated concentrations in the formulation, it was surprisingly having no additional IR bands interfering with the mentioned IR absorption bands belonging to LFX. The overlay spectra of excipient and API in Figure 1 mark no interference at the selected region for quantification [14, 20]. For further clarity of the possible interference, the spectra of each individual excipient and LFX were taken to establish the specificity of this proposed quantification method provided in Figures S1–S4. The complete spectral range spectra from 4000 to 700 cm^−1^ in Figure S5 also demonstrate that the standard addition technique did not evidence spectral interference due to concomitant absorbing species coming from excipients (Figure S6).

**FIGURE 2:**
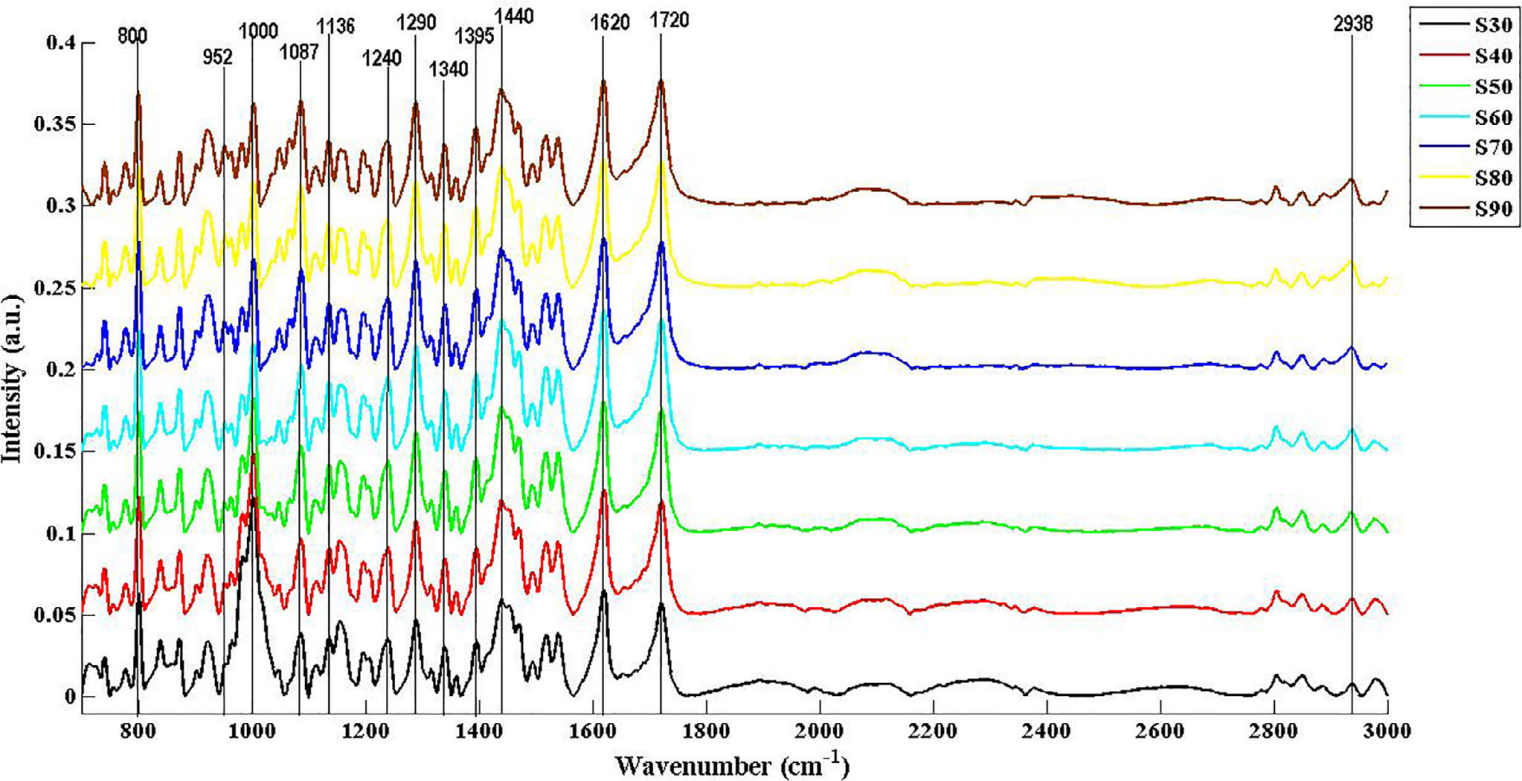
Concentration‐dependent response of FTIR spectra of solid mixture of levofloxacin API and excipients at different concentrations (SPL30–SPL90).

Although all these functional groups respond specifically for concentration‐dependent response of LFX that is given in Figure 2, most suitable region should be one that showed reliable response for all defined parameters of method development. The band selection for quantitative analysis should produce a strong peak, have significant selectivity and specificity for excipients and provide a linear response with increasing concentrations of calibrants. Some functional groups like C = O, C–N, C–F or COOH groups are the most commonly used for quantitative purposes because these functional groups exhibit strong, sharp and well‐resolved peaks. Strong peaks provide better sensitivity and sharp signal which enhance the specificity. So different calibration curves were made through selection of different functional regions at peak area as well as the peak height for calibration model, and their results are given in Table 3.

**TABLE 3:** A comparison of sensitivities of levofloxacin IR regions and frequencies with their linearity.

Spectral characteristic	Test region	Measurement mode	Regression line	Correlation coefficient *R* ^2^
Point 1	1617.7	Peak height	15.36*x* + 0.38	0.865
Point 2	1317.5	Peak height	17.14*x* + 0.35	0.798
Point 2	1238.5	Peak height	0.02*x* + 0.015	0.982
Point 2	1057.6	Peak height	10.16*x* + 1.03	0.785
Region 1	1252.39–1218.84	Peak area	3.475*x* + 0.253	0.9943*
Region 2	1349.30–1323.20	Peak area	2.004*x* + 0.2667	0.96385
Region 3	1405.21–1367.93	Peak area	4.219*x* + 0.061	0.9884
Region 4	1636.30–1584.12	Peak area	5.213*x* + 1.134	0.95002

*Note*: Value with “*” indicates the highest *R* ^2^ value, showing model performance of the region.

**FIGURE 3:**
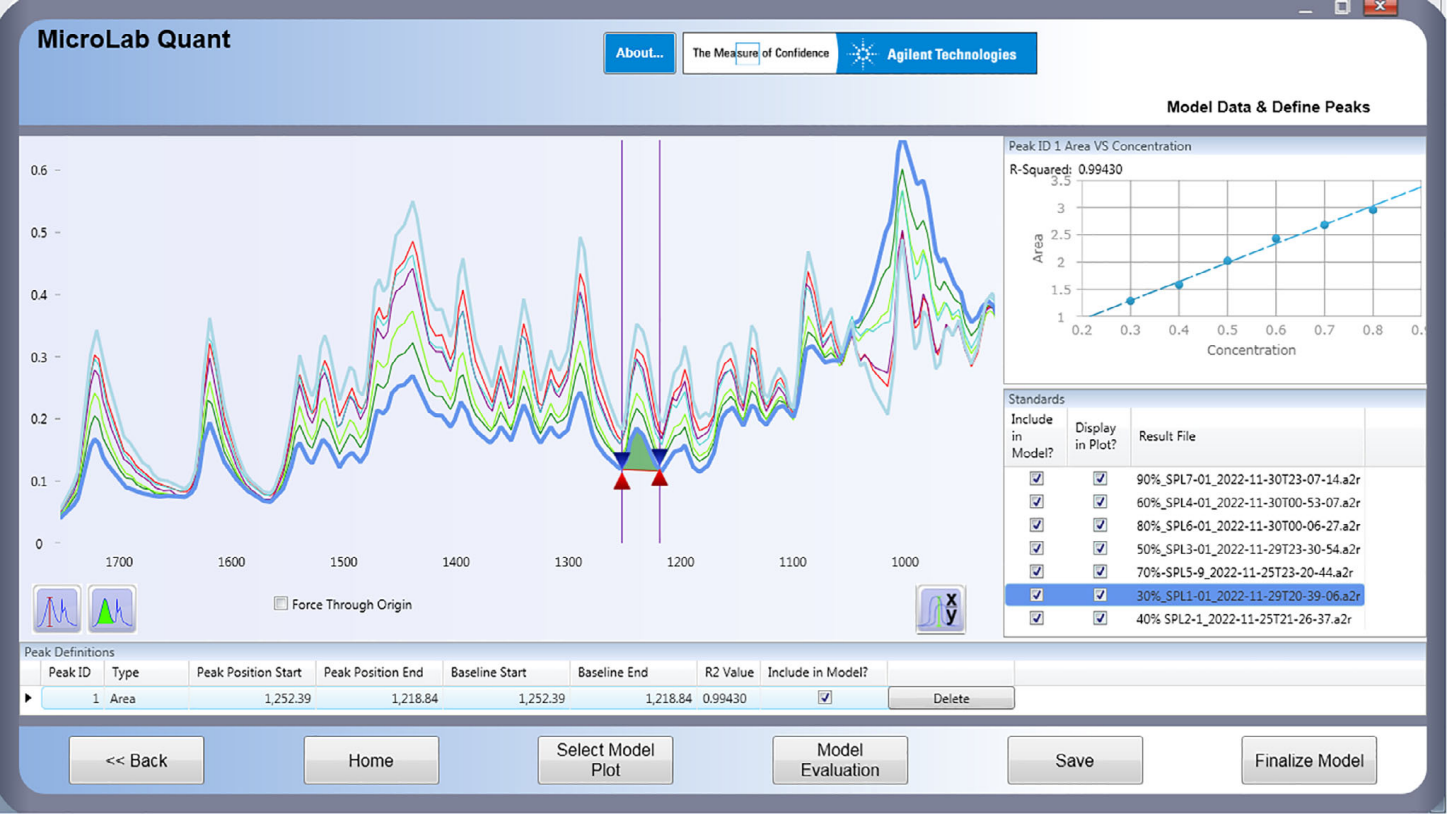
FTIR spectra of levofloxacin calibration standards from 30% to 90% w/w with selection region (1252–1218 cm^−1^) for quantification and regression model.

Interestingly, peak area showed better response in terms of linearity and robustness in comparison to peak height which suggests that selection of peak area at specific region could be the choice of model. Among the test regions, region 1 showed most reliable and reproducible results for calibration model and showed regression coefficient (*R* ^2^) value of 0.995 which is in acceptance range of ICH guidelines [21]. The selected region 1, which showed high molar absorptivity, is considered a stretching band of aromatic C–F and is a characteristic of LFX [22]. This group is uniquely present in LFX structure; hence, it does not frequently overlap with bands of other functional groups. Therefore, this functional group gives specificity to LFX chemical structure and can be selected for method development. Indeed, C–F stretching especially aromatic fluoro group produced sharp and strong IR frequency in the range of 1400–1200 cm^−1^. Therefore, considering the above information, the LFX spectrum showed notable frequency bands in the mid‐IR regions with maximum wavenumber located at 1252 and 1218 cm^−1^ which is selected for the quantification of LFX (Figure 3).

### 3.2 Analytical Figures of Merit for Method Validations

#### 3.2.1 Linearity, LOD and Quantification

Simple regression line was drawn at the selected region of 1218 and 1252 cm^−1^ for response of IR absorbance with range of concentrations as described in Table 1, which demonstrated acceptable linearity over 30%–90% w/w of the concentration range. The *y*‐intercept was slightly deviated from zero origin but not significantly different from zero, *p* < 0.05. Furthermore, the *y*‐intercept variability resulted less than 1% of the response obtained at the target level which is usually an acceptable criterion [23]. The statistical significance of the regression line (*R* ^2^ > 0.995) also revealed workability of slope, *p* < 0.05, which, in turn, suggested that changes in the concentration variable are highly associated with changes in the response variable. The LOD represents the minimum quantity of a substance that can be distinguished, and LOQ is defined as the smallest substance that can be quantified [24]. LOD of the test method was calculated through linearity of calibration curve and determined as 7.616% w/w. The LOQ was measured through ICH guidelines and calculated as 23.079% w/w, and results are described in Table 4 [25].

**TABLE 4:** Analytical method validation parameters based on the calibration curve for levofloxacin quantification.

Analytical parameter	levofloxacin
Linearity range (%)	30%–90% w/w
Coefficient of determination (*R* ^2^)	0.9943
Coefficient of correlation (*R* ^2^)	0.9952
Equation of a straight line	*y* = 3.475*x* + 0.2527
Intercept (a) ± SD	0.0227 ± 0.0120
LOD	7.616% w/w
LOQ	23.079% w/w

Abbreviations: LOD, limit of detection; LOQ, limit of quantification.

#### 3.2.2 Precision

The precision of developed method was calculated by using three different levels of concentrations: low (30% w/w), medium (50% w/w) and high (70% w/w) of calibration concentrations. The precision of the method was ensured through repeatability on an external test with six replicates, and relative standard deviation was measured [26]. Although all test concentrations showed good repeatability, having % RSD <1, which strongly agreed the acceptance criteria ≤2% RSD. However, among the test concentrations, low level of concentration showed good repeatability (0.652% RSD). Intermediate precisions were assured through inter‐day and intraday precisions with two different analysts. Both tests were conducted at five replicates which produced % RSD of 0.852 and 0.451, respectively, which is significantly lower than accepted range ≤2% RSD. As can be observed from Table 5, data for repeatability and instrument precision met the requirement of method to be precise for quantification of LFX in solid dosage form [27]. Our findings were agreed with reports of FTIR quantification and method validation for solid dosage form [26, 28].

**TABLE 5:** The Precision result of ATR–Fourier transformed infrared spectroscopy (FTIR)‐based method.

Parameter	Level	Concentration	Observation	% RSD	Acceptance criteria
Repeatability, *n* = 6	Low	30% w/w	30.7% w/w	1.215	≤2% RSD
	Medium	50% w/w	51.2% w/w	0.85	
	High	70% w/w	68.9% w/w	1.14	
Inter‐day operator 1, *n* = 10	Medium	50% w/w	49.8% w/w	0.69	≤1% RSD
Inter‐day operator 2, *n* = 10		50% w/w	50.7% w/w	0.88	
Intraday precision, *n* = 5	Medium	50% w/w	49.5% w/w	1.21	≤2% RSD

#### 3.2.3 Accuracy

The ICH defines accuracy as proximity of data obtained by developed method to actual value. The accuracy and efficiency of the test method were assured through recovery study. The recovery study was conducted by standard addition method (spiking of standard), and amount was measured through chemometric model [29]. Interestingly, all the results of recovered amount were in good acceptance limit determined through % RSD and found <1.2% of added amount, and results are described in Table 6. The mean recovery percent values ranging from 99.30 to 100.91 were obtained, indicating high accuracy of the proposed method. The recovery percent values are within the range specified by AOAC [30].

**TABLE 6:** Percentage accuracy of levofloxacin on ATR–Fourier transformed infrared spectroscopy (FTIR).

Theoretical conc. % w/w	Experimental conc. % w/w	%Accuracy	% RSD
60.96	61.2	100.39	0.7412
76.20	76.9	100.91	0.6524
91.44	90.80	99.30	0.4517

#### 3.2.4 Chemometric Model

**FIGURE 4:**
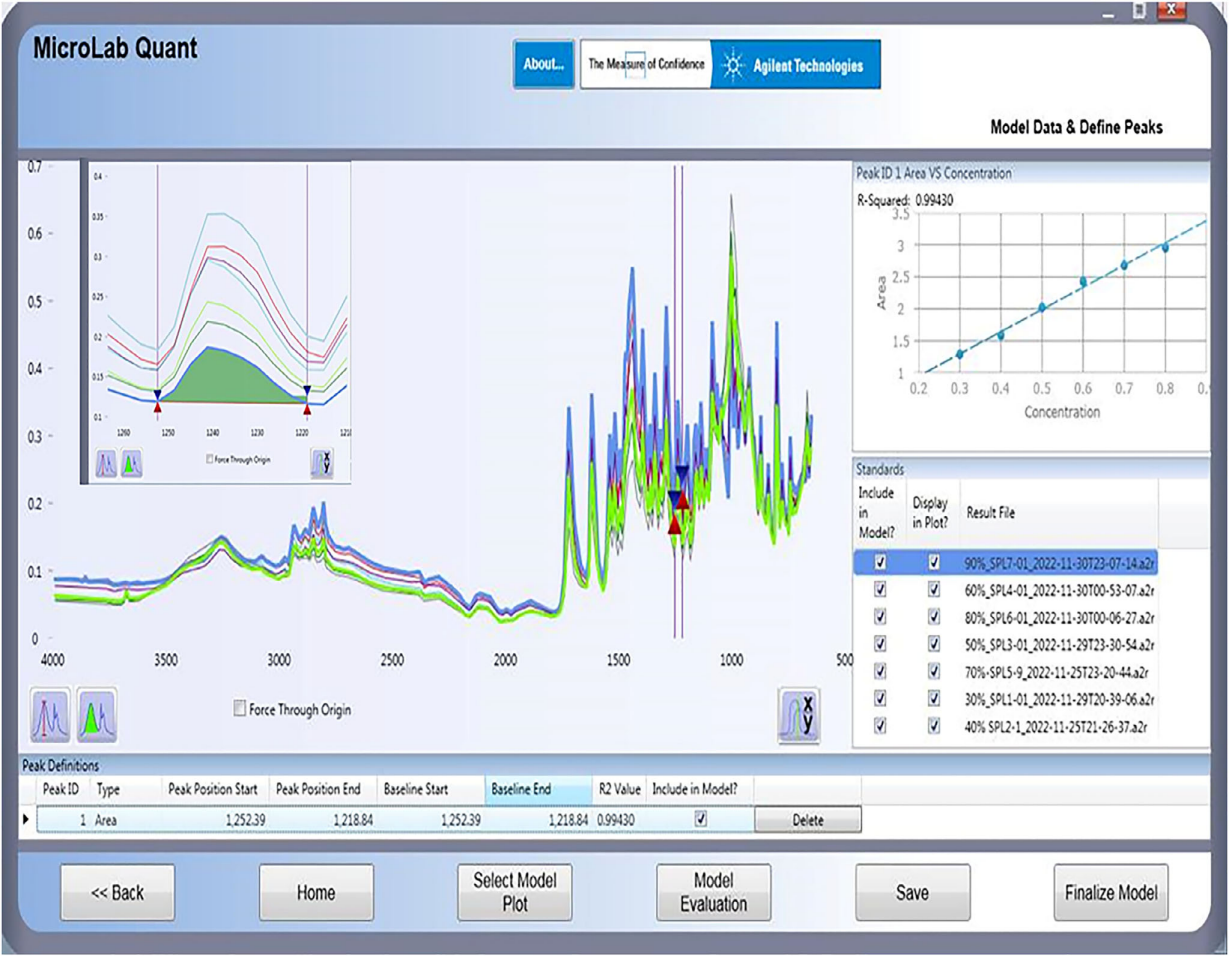
Chemometric model for calibration curve and quantification at specific area valley (1252–1218 cm^−1^).

The quantification model was designed chemometrically using micro lab quant software. In this model, a series of calibration standards of different concentrations (30%–90% w/w) were used to build a calibration curve model. All the calibration standards were scanned in the region 4000–400 cm^−1^, and the area under the curve of selected region was used for development of analytical model. The calibration curve achieved with the regression coefficient of *R* ^2^ = 0.995 under the curved area at 1252–1218 cm^−1^. In Figure 4, designed chemometric model showed good linearity in the selected region with regression equation of line *y* = 3.475*x* + 0.2527 and obtained linearity in line agreement to ICH and AOAC guidelines for the development of method.

This chemometric model was used for identification and assays of test samples (blind samples). The assay of LFX tablets by chemometric model of ATR–FTIR was conducted without extracting it in any solvent from its dosage form. The method requires the finely ground powder and applied directly on the lens of ATR–FTIR and scanned for spectrum which is arithmetically calculated by chemometric model through software. The assay values of test samples are measured directly, and results are given in Table 7.

**TABLE 7:** The result of different brands tested with new developed method.

Sample	Levofloxacin (mg)	FTIR	HPLC	% RSD
Brand 1 Leflox 500 mg	500	506.2 ± 0.31	501.5 ± 0.56	0.660
Brand 2 Floxolev 500 mg	500	502.0 ± 0.28	498.1 ± 0.18	0.551
Brand 3 Curafloxain 500 mg	500	505.7 ± 0.08	501.8 ± 0.46	0.547

Abbreviation: FTIR, Fourier transformed infrared spectroscopy.

**FIGURE 5:**
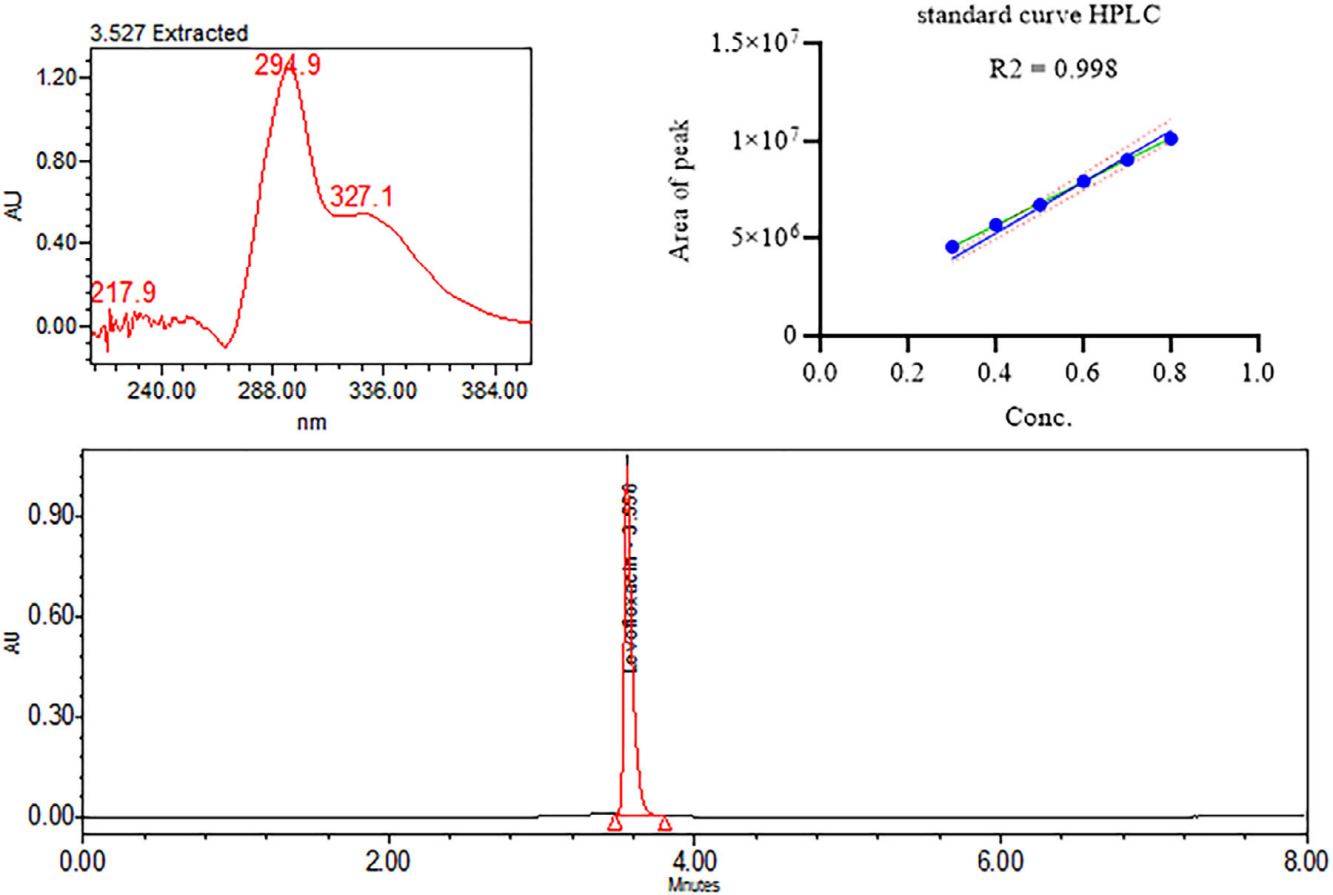
HPLC‐PDA analysis of levofloxacin standards (0.20–0.90 mg mL^−1^) and unknown determinations.

The validation of the results was achieved through comparison of assay values measured through HPLC‐PDA. Assay values measured through HPLC were <2% RSD from assay values of same samples measured through FTIR‐based test method, and results are given in Table 7. *t*‐Test values indicate that test method showed good trueness of measured values and validated through HPLC‐PDA (Figure 5).

#### 3.2.5 Partial Least Squares Regression Analysis (PLSR)

The presence of adulterants in pure sample can be easily detected through PCA plot through positive and negative loadings while accurately comparing with IR spectra of pure samples. Indeed, FTIR spectroscopy highlighted the slight variations in spectral behaviour that are associated with changes in functional groups but hardly quantified through PCA plot [31]. In order to build a predictive quantitative model for different solid dosages of LFX the PLSR model was built in consideration of latent variables (factors) which comes a best fit for chemometric indicators. This PLS regression model for LFX was designed with seven different concentrations of LFX (SPL30–SPL90), whereas fitness of model was assured through chemometric model indicators *R* ^2^ and RMSE values [32]. RMSE value indicates the absolute fit of the model to the data, whereas *R* ^2^ indicates the linearity of the obtained regression of test model [33]. Cross validation of the built model was confirmed with RMSEP value, another indicator which predicts the accuracy of external test (difference among real and estimated values) [34]. The spectral data of test samples were processed without applying vector normalization algorithm. The construction of a predictive model is the first step of PLSR analysis through reference samples with known quantity of LFX (SPL30–SPL90) to predict unknown concentration of sample.

**FIGURE 6:**
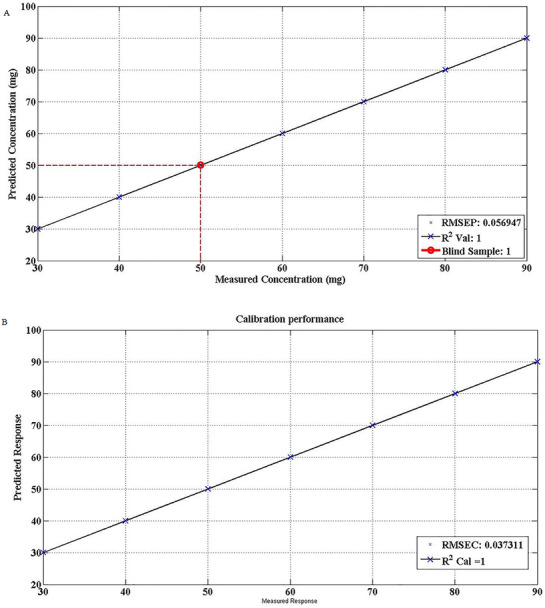
Performance (calibration vs. prediction) of the PSLR model for FTIR multivariate spectral data for the quantification of levofloxacin in solid dosage form: (A) calibration curve and (B) quantification of blind sample.

The determination coefficient of calibration *R* ^2^ (cal) and the prediction for blind sample (*R* ^2^ Val) were found to be one which shows the acceptability and efficiency of the build model for quantification of test samples. The root mean square values were found slightly low (RMSC = 0.056947) for the calibration data which showed good predicative performance and robustness of the model. The built PLRS model of test method was employed for quantification of blind sample (SPL50) having LFX 50% w/w in given dosage form. This model estimated the blind sample of 50.8% ± 0.2% w/w of LFX, and results are shown as red line in graph, Figure 6A. The estimation of blind sample was achieved through using each spectrum separately that projected the response on designed model for prediction of unknown concentration. The results obtained from prediction model are presented as mean ± standard deviation. The predicted values of blind samples were cross validated by comparing results with analysis of same sample through approved method of HPLC to consolidate the predictive capacity of PLS models. Interestingly, the results obtained from test method were very comparable with findings of standard method, and results are given in Table 3. The calibration and prediction results of the developed PLS FTIR method were given in Table 7.

#### 3.2.6 Principal Component Analysis

The PCA is used in molecular spectroscopy which converts original variables to multivariable data and generates new variables called principal components. When these variables (PCs) plot, it showed response for even minor variations. The new PCs could be in direction to original variable like PC1 that refers to the direction having maximum variance, whereas PC2 refers to direction for second most variance [35]. Each variable of test sample can be plotted through new coordinate system that originates loading and score plots [36]. In the present study, score plot through PCA was originated and used to evaluate impact of adulterant concentrations on the IR spectrum of active substance. This score plot can also identify minor variations on IR spectrum of LFX by any means via degradation or adulteration during storage and manufacturing. In the selected model, when API blended S6 and commercial product of LFX tablets were compared, PC1 and PC2 explained 99.93% and 99.91% of IR spectral variance.

**FIGURE 7:**
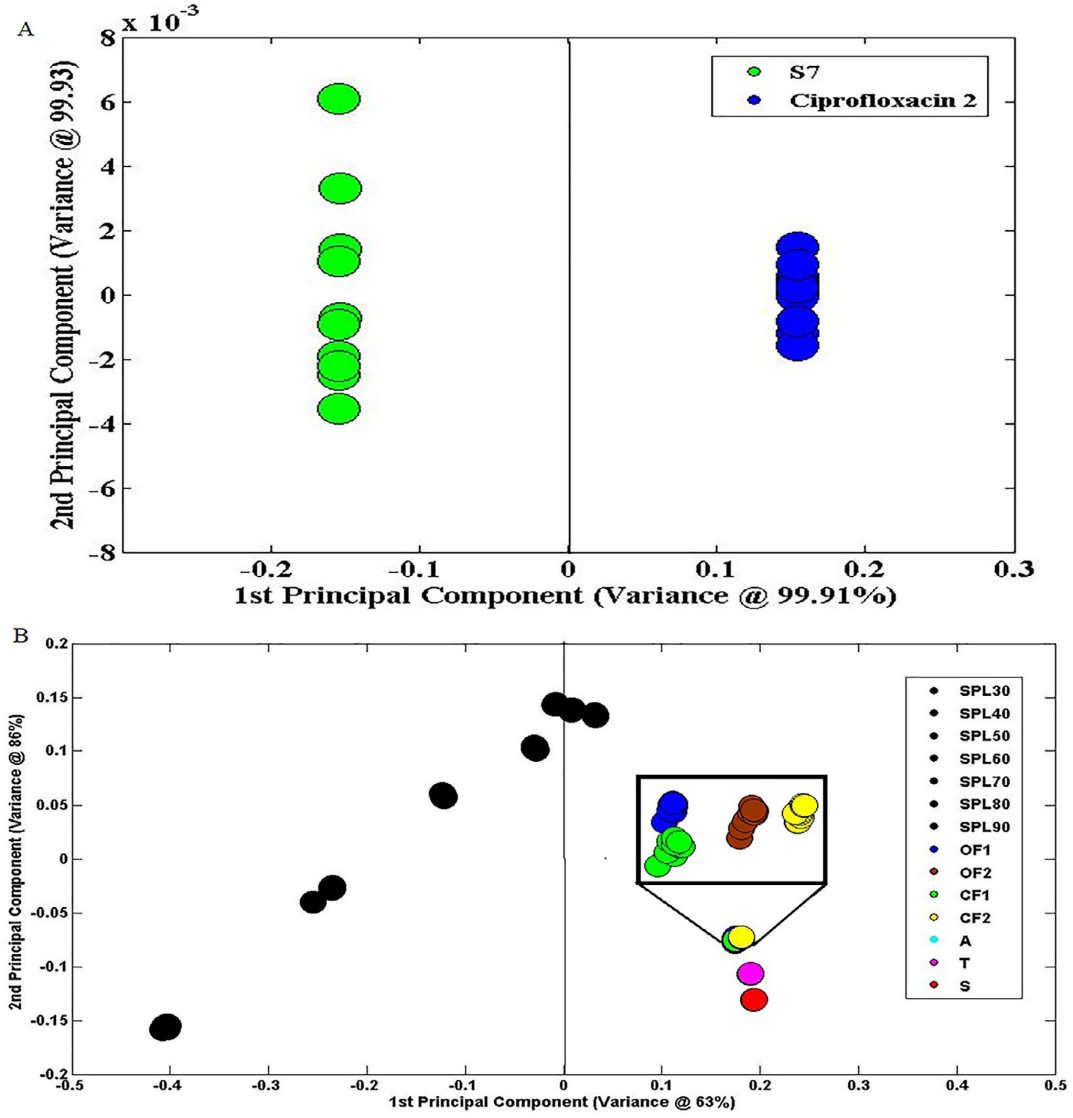
(A) selected plot of the best PCA model for the discrimination of adulteration of ciprofloxacin in test sample from pure sample (levofloxacin alone), (B) plot of PCA model of calibrated standards (SPL30–SPL90) and adulterated samples (OF and CF) and forced degradation sample (A, T and S).

The scatter plot of FTIR spectral data for adulteration or degradation (forced degraded samples) was significantly differentiated from pure commercial samples, and results are presented in Figure 5. The score plot showed PC1 variance of 63% and PC2 variance of 86% for adulterated samples (OF1–OF2, CF1–CF2) and degraded samples (A, T and S). These two principal components, first of all, showed good discriminations among pure sample and adulterations, and a trend related to adulterant concentration was clearly evident by PC1. Figure 7 illustrates that PC1 accounted for 63% of total variance that was successfully able to separate pure sample from adulterated sample on the basis of percentage adulteration. Indeed, pure samples were located on the left of quadrant, whereas adulterants were found on the right of quadrants. The position of adulterants was shifted to increasing value of PC1 by slight increase in percentage of impurities in pure sample which indicates estimation of impurity in pure sample as well as strength of impurity in the sample mixture. Interestingly, the impurity of ofloxacin in pure sample was significantly differentiated by PC1 by showing OF1 at 0.12 positions while 0.23 position for OF2 at right quadrant which found near zero for PC2. But, when stability samples (forced degradation), A, S and T were studied in score plot; second component (PC2) clearly indicates differentiation from pure sample which accounts for 86% of variance, whereas PC1 showed constant score. The clarification of this sample distribution in score plot at various quadrants of PC1 and PC2 lies in the chemical composition of impure samples.

## 4 Conclusion

ATR–FTIR spectroscopic method is based on simple and rapid quantification for routine drug quality control analysis. Despite interference caused by the presence of excipients in dosage matrix, the method is still cost‐effective, free from laborious sample preparation and environmentally friendly. The method demonstrated through its results that it is equally useful to quantify LFX in raw material and API in solid dosage forms without any sample preparation by the analyst. In addition to assay of active substance, the developed FTIR method, when coupled with PSLR and PCA tools, was able to determine any possible adulteration or degradation along with quantification of LFX. The specificity and selectivity assured that method was robust for quantification of LFX. Recovery analysis confirmed the repeatability and reproducibility of assay value, whereas <2% RSD from results measured by HPLC‐based assay method. This regression model has shown that the method offers ease of analysis as compared to traditional HPLC method. FTIR equipment is a crucial tool in pharmaceutical industry or regulatory bodies for qualitative purposes but still undervalued for quantitative analysis. The proposed FTIR‐based method, in comparison with compendial and non‐compendial analytical methods, eliminates the solvent or reagent consumption and offered cost‐effective and quick method. Finally, by selecting different measurement spectral bands, this study opens up the possibility of applying the proposed FTIR method to quantify LFX when combined with other API in the same dosage form.

## Author Contributions

**Muhammad Kashif**: conceptualization (lead), formal analysis (lead), software (lead), writing – review and editing (equal). **Muhammad Imran**: resources, writing – review and editing (equal). **Rizwan Ashraf**: project administration (lead), methodology (lead), conceptualization (supporting), writing – original draft (supporting), writing – review and editing (equal). **Muhammad Umair Kamal**: writing – original draft (lead), data curation (lead), methodology (supporting), **Raja Adil Sarfraz**: validation (lead), review and editing (equal).

## Conflicts of Interest

The authors declare no conflicts of interest.

## Supporting information




**Supporting File 1**: ansa70048‐sup‐0001‐SupMat.docx

## Data Availability

The data that support the findings of this study are available on request from the corresponding author. The data are not publicly available due to privacy or ethical restrictions.
