# Supplementary material for: ATR–FTIR‐Based Direct and Rapid Quantification of Levofloxacin, Degradation and Adulterations via Multivariate Analysis
Source: Anal Sci Adv. 2025 Oct 3;6(2):e70048. doi: 10.1002/ansa.70048 (PMC12491855; doi:10.1002/ansa.70048)
Supplement: Supplementary file 1 — Supporting File 1: ansa70048‐sup‐0001‐SupMat.docx [file ANSA-6-e70048-s001.docx]

**ATR-FTIR based Direct and Rapid Quantification of Levofloxacin, degradation and adulterations via Multivariate analysis**

Muhammad Umair Kamal^1^, Raja Adil Sarfraz^1^_,_ Rizwan Ashraf^1^**, Muhammad Kashif^2^*, Muhammad Imran^3^
1-Depratment of Chemistry, University of Agriculture Faisalabad, Pakistan

2-TU Dublin-FOCAS Research institute, Aungier street, Dublin 2, D02 HW71, Ireland

3- National Institute for Biotechnology & Genetic Engineering (NIBGE), Faisalabad-Pakistan

Corresponding author: [Muhammad.Kashif@tudublin.ie](mailto:Muhammad.Kashif@tudublin.ie), ** [rizi_chem82@hotmail.com](mailto:rizi_chem82@hotmail.com)

**Figure S1: FTIR Overlay spectrum of Levofloxacin API (Light Violet) and Talcum Powder (green) in the region of 2000cm^-1^-1000cm^-1^ exhibits no matrix interference in the selected region.**

**Figure S2: FTIR Overlay spectrum of Levofloxacin API (Light Violet) and Avicel (Dark Blue) in the region of 2000cm-1-1000cm-1 exhibits no matrix interference in the selected region.**


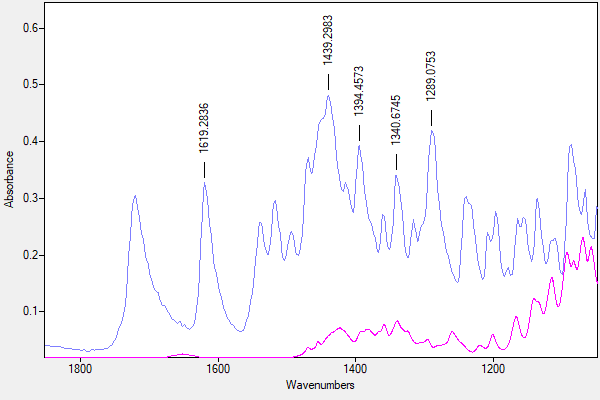


Lactose Monohydrate

Levofloxacin

**Figure S3: FTIR Overlay spectrum of Levofloxacin API (Light Violet) and Lactose Monohydrate (Pink) in the region of 2000cm-1-1000cm-1 exhibits no matrix interference in the selected region.**


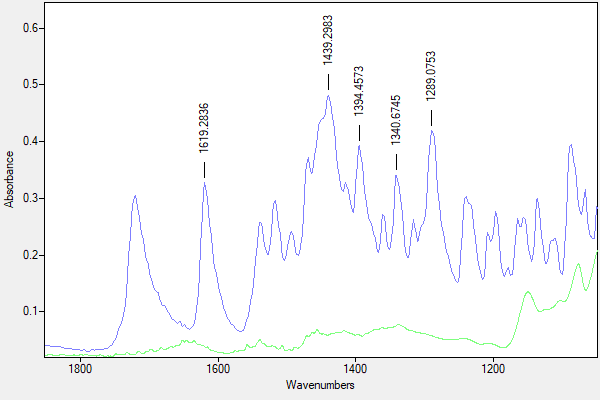


Levofloxacin

Starch Powder

**Figure S4: FTIR Overlay spectrum of Levofloxacin API (Light Violet) and Starch Powder (Light green) in the region of 2000cm-1-1000cm-1 exhibits no matrix interference in the selected region.**

**Fig S5: Overlay of All excipients and API over the complete spectral range 4000 cm^-1^ to ~700 cm^-1^ which exhibits no interference in the selected region for quantification.**


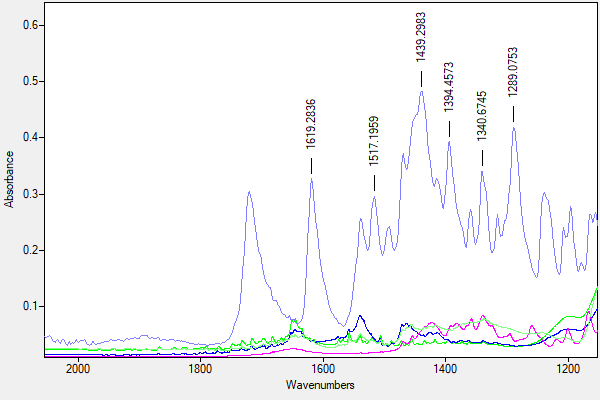


-Levofloxacin

-Talcum Powder

-Avicel

-Lactose Monohydrate

-Starch Powder

**Fig S6: Overlay Spectra of API and all excipients from the region 2000 cm^-1^ to 1000 cm^-1^ which exhibits no interference in the selected region for quantification.**


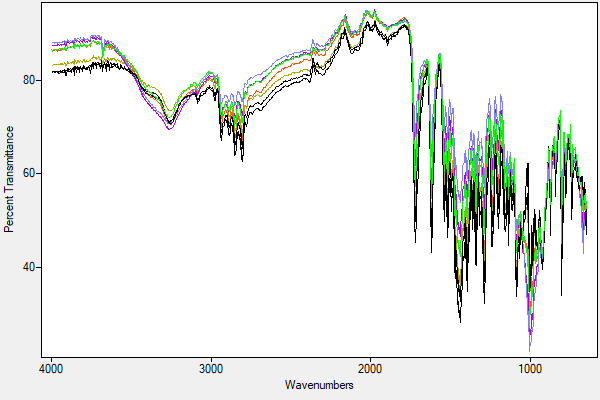


**Fig S7: Overlay of Transmission FTIR Spectra of Calibration standards from 30% to 90% acquired by Agilent 630-ATR-FTIR Spectrophotometer.**
